# The Use of Polymer Chitosan in Intravesical Treatment of Urinary Bladder Cancer and Infections

**DOI:** 10.3390/polym10030265

**Published:** 2018-03-05

**Authors:** Andreja Erman, Peter Veranič

**Affiliations:** Institute of Cell Biology, Faculty of Medicine, University of Ljubljana, Ljubljana 1000, Slovenia; andreja.erman@mf.uni-lj.si

**Keywords:** chitosan, biological activity, medical applications

## Abstract

The most frequent diseases of the urinary bladder are bacterial infections and bladder cancers. For both diseases, very high recurrence rates are characteristic: 50–80% for bladder cancer and more than 50% for bladder infections, causing loss of millions of dollars per year for medical treatment and sick leave. Despite years of searching for better treatment, the prevalence of bladder infections and bladder cancer remains unchanged and is even increasing in recent years. Very encouraging results in treatment of both diseases recently culminated from studies combining biopolymer chitosan with immunotherapy, and chitosan with antibiotics for treatment of bladder cancer and cystitis, respectably. In both pathways of research, the discoveries involving chitosan reached a successful long-lasting cure. The property of chitosan that boosted the effectivity of illness-specific drugs is its ability to enhance the accessibility of these drugs to the very sources of both pathologies that individual treatments without chitosan failed to achieve. Chitosan can thus be recognised as a very promising co-player in treatment of bladder cancer and bacterial cystitis.

## 1. Urinary Bladder Function and Malfunctions

**Figure 1:**
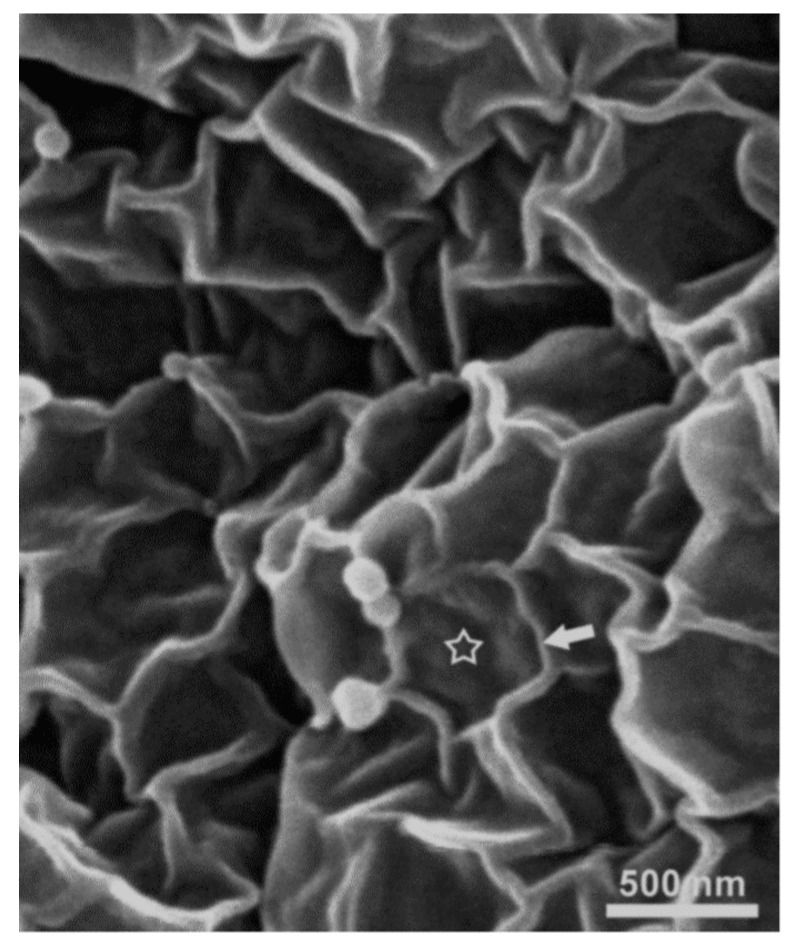
Apical plasma membrane of umbrella cell. The scalloped appearance is due to urothelial plaques (asterisk) of thickened membrane surrounded by hinge regions of normal membrane (arrow), as seen under the scanning electron microscope.

The main function of the urinary bladder is to enable controlled micturition by retaining the urine at constant pressure between periodic micturition. To prevent the permeation of potentially toxic and hypertonic urine back into underlying tissues and bloodstream during the retention, the luminal side of the urinary bladder is covered by a three-layered epithelium called the urothelium [1]. The superficial urothelial cells, also called umbrella cells, are mainly responsible for maintaining a blood-urine barrier, which is supposed to be the tightest among the barriers in mammalian tissues [2]. These cells have a highly specialised apical plasma membrane [3] composed of proteins uroplakins, which are arranged in semi-crystalline structures forming rigid-looking urothelial plaques of thickened membrane, interspersed by normally thick membrane areas called hinge regions, altogether give apical plasma membrane a characteristic scalloped appearance (Figure 1) [4]. The permeation of metabolites in the urine is additionally prevented by a thick layer of glycocalix [5], while a paracellular diffusion of the urine is limited by tight junctions [6,7]. 

To maintain the barrier function in spite of constant changes of urine volume in the bladder, the umbrella cells have to adjust to extreme changes of the surface between filled and empty bladder [8,9] by reversible adjustment of apical surface and by altering their shape from cuboidal to extremely flat [10]. In order to prevent damage of umbrella cells during the filling of the bladder and distension of the urothelium, the subapical actin cortex, widely present in most epithelial cells, is in umbrella cells replaced by a compact cytokeratin network in the same location [11,12]. This provides cells with a much stronger mechanical support. The cytokeratin network is connected to a dense array of desmosomes concentrated in the subapical area of lateral membranes [13]. Replacement of superficial urothelial cells occurs constantly during the renewal of the urothelium, but the turnover of cells in the normal urothelium is very slow, because the life span of umbrella cells may be 6–7 months in physiological conditions [14]. However, after injury, a proliferation of basal cells increases rapidly, leading to fast renewal of intermediate and umbrella cells and the reestablishment of the permeability barrier function [15,16].

Such a stable epithelium is well adapted for maintaining the barrier function. However, this characteristic of the urothelium is largely responsible also for the limited success in treatment of the most frequent pathologies of the urinary bladder, bladder cancer and bacterial cystitis, because the blood-urine barrier prevents medications from reaching cancer cells in deeper layers of the urothelium and the bacteria hidden inside urothelial cells. To improve the penetration of drugs into urothelial tissue, several inducers of urothelial cell desquamation could be applied. Among them, mainly cyclophosphamide [12,17], 12-o-tetradecanoylphorbol-13-acetate (TPA) [18], sodium saccharin [19], or hyperthermic shock [20] have been used to largely remove urothelial cells. Most procedures that induced cell removal resulted in an inflammatory response, prolonged cell desquamation, and transitional hyperplasia of the urothelium. The most successful way to enhance the permeability of urothelium with minimal inflammatory response to the neighbouring tissue was proven to be the application of biopolymer chitosan that can be used for the controlled removal of the superficial layer of urothelial cells with minimum damage to the rest of the bladder wall [21].

## 2. Effect of Chitosan to the Urothelium 

Chitosan, a cationic polysaccharide composed of glucosamine and *N*-acetyl glucosamine, is obtained by partial deacetylation of chitin. In general, chitosan is regarded as a biodegradable, biocompatible nontoxic polymer [22]. Chitosan has been widely used in biotechnology and reconstructive medicine [23]. Surprisingly, in the urothelium, chitosan (chitosan hydrochloride, 86% degree of deacetylation, 30–400 kDa) causes a very rapid drop in transepithelial resistance, indicating for the effect on tight junctions that was proven in both ex vivo [16] and in vitro experiments [24]. However, the mechanisms responsible for chitosan activity are largely unknown, even though they are essential for the proper use of this biopolymer. It has been proposed that the mechanism for an interruption of epithelial barrier function is mainly due to electrostatic interaction between the positively charged chitosan (85% degree of deacetylation, 80 kDa) and the negatively charged integrin α(V)β(3). This electrostatic interaction can lead to the conformational change of integrin α(V)β(3) and its clustering along the cell border, F-actin reorganization, and claudin 4 down-regulation, eventually resulting in the disruption of tight junctions and consequent increase of paracellular permeability [25]. Another mechanism by which chitosan (15% degree of deacetylation, *M*_n_ = 108,700) increases the permeability of the epithelia is by its interactions with cell membranes. The high density of positive charges is responsible for chitosan to interact strongly with negatively charged [26] and neutral molecules [27,28] in the plasma membrane. The combination of electrostatic and hydrophobic interactions can be crucial for binding of chitosan to phospholipids at the plasma membrane surface [26], and when chitosan is able to enter deeper into the lipid bilayer, the molecules can interact by hydrophobic interactions also with membrane fatty acids. Extraction of cholesterol has been proposed as a major mechanism of chitosan action, however, studies of the interaction between chitosan and membrane models proved that chitosan only binds to cholesterol in membranes but cannot remove this molecule from the membrane [29]. Protonated amino groups of chitosan enable the polymer to interact also with a negatively charged mucus molecules covering the epithelia via electrostatic interactions [30]. A higher charge density is obtained at acidic pH values, since the p*K*_a_ value of the d-glucosamine residue of chitosan is about 6.2–7.0 [31]. The charge density of chitosan is therefore considered to be an important factor in the drug absorption enhancement caused by this polymer. 

**Figure 2:**
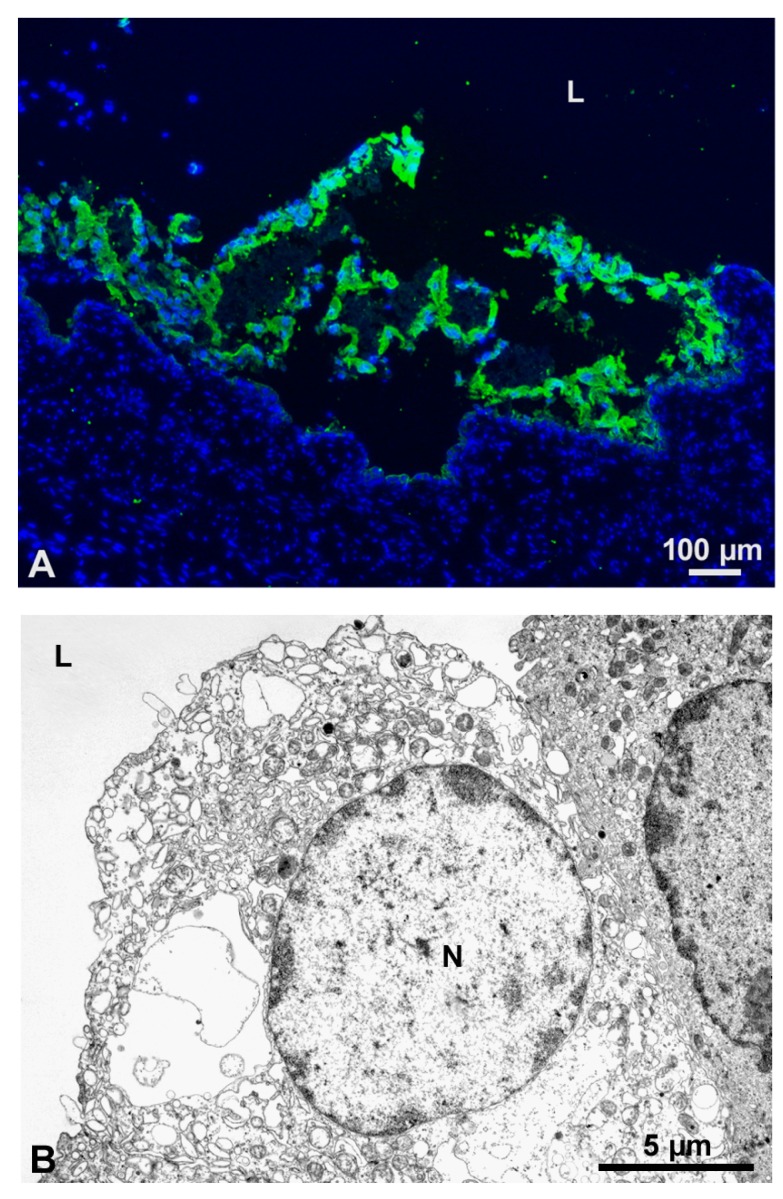
Chitosan induced umbrella cells desquamation (A) and necrosis (B). (**A**) Cytokeratin 20-positive umbrella cells (green fluorescence) detach from the urothelium after intravesical application of chitosan. (**B**) These cells have distinctive signs of necrosis under transmission electron microscope. *N*-nucleus, l-urinary bladder lumen.

Intravesical application of chitosan (chitosan hydrochloride, 86% degree of deacetylation, 30–400 kDa) to experimental animals resulted in induction of urothelial cell desquamation [15] (Figure 2A). However, the precise mechanism of induction of desquamation is still poorly understood, and further investigations must therefore be performed to explain the mechanism by which chitosan acts on urothelial cells. It has been discovered that chitosan adheres to the apical membrane of umbrella cells and causes necrotic changes and desquamation of superficial cells (Figure 2B). The apical plasma membrane of terminally differentiated superficial cells is composed of rigid urothelial plaques and normally thick membrane areas [32]. The main constituents of urothelial plaques are uroplakins, which are highly glycosylated proteins contributing to the urothelial glycocalyx [33]. Due to cationic nature of chitosan, this polymer can adhere to negatively charged groups of glycosaminoglycan in plaque regions, as well as to hinge regions of the apical plasma membrane of superficial cells. It is therefore possible that breaks in the apical membrane appear because of different viscoelastic properties of the discrete domains, to which the chitosan molecules are attached, which is in agreement with the finding of Pavinatto et al. [26]. When covered by a chitosan layer, the breaks in the apical plasma membrane can appear because of repeated stretching and contracting of the elastically and structurally inhomogeneous apical plasma membrane of umbrella cells. At higher chitosan concentrations (chitosan hydrochloride, 86% degree of deacetylation, 30–400 kDa) and longer exposure times, the release of cellular content and lysosomal enzymes from umbrella cells subsequently triggers cell death of also underlying urothelial cells [21].

It has been well documented that by choosing the appropriate chitosan concentration and the duration of intravesical application, chitosan can induce a controlled desquamation of urothelial cells in experimental animals. Intravesical application of 0.005% chitosan dispersion (chitosan hydrochloride, 86% degree of deacetylation, 30–400 kDa) caused complete removal of exclusively superficial cells after 20 min of treatment. A complete regeneration of this mild injury was completed within 60 min after an application of chitosan, and no inflammatory cell response was determined in such a short period of time. In addition to the morphological characteristics of the regeneration process after the chitosan-induced disruption of the urothelium, functional regeneration was also shown as a rapid restoration of the transepithelial resistance of urothelial tissue. By in vivo and ex vivo experiments, such intravesical chitosan application was proven to be safe in experimental animals because only weak inflammatory response and no persistent urothelial damage were detected [16].

## 3. Chitosan in Treatment of Urinary Bladder Cancer

Bladder cancer is the fourth most common cancer in males. Worldwide annual incidence of bladder cancer is estimated to be 14.1 million cancer cases around the world in 2012 [34,35]. The muscle non-invasive bladder cancer, which is the most common cancer of urinary bladder, usually appears as multiple focal tumours distributed throughout the urothelium of the bladder [36]. Urothelial tumours are treated depending on the metastatic potential with transurethral tumour resection (TUR) for non-invasive papillary tumours, and with cystectomy for invasive tumours, followed by radiation treatment or chemotherapy, or immunotherapy. In urothelial tumours, the recurrence rate is 50–80%, being the highest of any major malignancy [37]. Despite advantages of local delivery that overcome systemic adverse effects, intravesical therapy has its limitations, mainly because of the blood-urine barrier limits the penetration of cytostatics to deeper layers of the urothelium. Reduced doses of cytostatic in deeper layers of the urothelium may preserve individual cancer cells as seeds for the growth of new tumours, which make frequent use of invasive cystoscopy necessary. It is thus an urgent need for further development [38] in which mainly investigative work with nanoparticles (NPs) appear to be a promising strategy for improvement [39] in both diagnostics and treatment. As early diagnosis of recurrent tumours is important, a recent development of peptide-targeted glycol chitosan nanoparticles containing ferromagnetic iron oxide for multimodal imaging became a promising tool for non-invasive detection of bladder tumours by magnetic resonance imaging and near infrared fluorescent imaging [40]. NPs are proposed also to be used in the treatment of bladder cancers. Namely, encapsulation of anti-tumour drugs into NPs can protect the drug from degradation, enhance its solubility, and enable controlled release in cancerous tissue [41]. Chitosan functionalisation has been proven to increase transurothelial penetration and tumour cell uptake of commonly used poly(lactic-*co*-glycolic acid) (PLGA) nanoparticles [42]. Chitosan-functionalized nanoparticles demonstrated an increased binding to and uptake in intravesically instilled mouse bladders at 10 times higher level in comparison to PLGA-only nanoparticles. Furthermore, binding a survivin siRNA to chitosan-functionalised nanoparticles significantly decreases the survivin expression and by that decreases the proliferation of bladder cancer cells. Thus, chitosan-functionalised nanoparticles proved to have the capacity to transport large amounts of siRNA across the urothelium and to the tumour site, thus increasing therapeutic response. 

**Figure 3:**
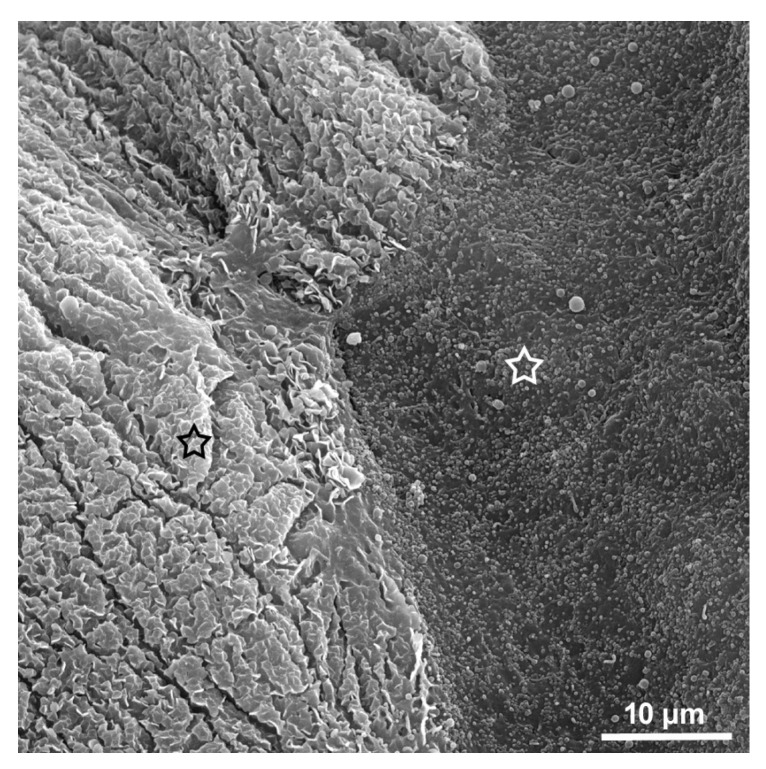
Apical plasma membrane of normal and cancer urothelial cells. Distinctive difference between scalloped apical membrane of normal umbrella cells on the left (black rimmed asterisk) and microvillar apical membrane of cancer cells on the right side (white rimmed asterisk) of the micrograph taken by scanning electron microscope.

In spite of encouraging results of using nanoparticles covered with chitosan, the penetration in deeper layers of the urothelium is still questionable, especially in the case of individual cancer cells migrating from the primary tumours below normal umbrella cells [43]. The most efficient way to temporarily compromise the blood-urine barrier appears to be the controlled removal of umbrella cells with chitosan. In our recent in vitro study, it became evident that chitosan selectively removes highly differentiated urothelial cells and is much less toxic to less differentiated urothelial cells [24]. The higher toxicity of chitosan to umbrella cells versus urothelial cancer cells and less differentiated urothelial cells of lower urothelial layers can be explained by existence of very specific scalloped apical plasma membrane only in umbrella cells but not in less differentiated normal or cancer urothelial cells (Figure 3). Specific toxicity of chitosan to umbrella cells provides an ideal tool to briefly enhance the access of anti-tumour agents to the nests of bladder cancer cells in deeper urothelial layers while not causing major obliteration of the urothelium [15]. 

**Figure 4:**
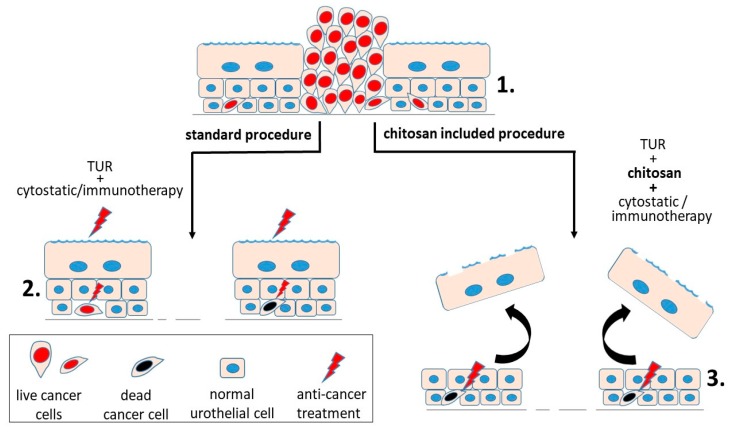
Two procedures to treat non-invasive urinary bladder cancer. (**1**) Urinary bladder cancer cells persist in the urothelium in tumours and as individual migrating cancer cells. (**2**) Standard procedure in treatment of non-invasive urothelial tumours is transurethral resection (TUR) of tumours and subsequent chemo- or immunotherapy. Frequently, individual cancer cells survive such treatment because of the diffusion-limiting blood-urine barrier. (**3**) Combined treatment with TUR removing tumour mass, chitosan compromising the barrier function, and immunotherapy more efficiently destroys even remote cancer cells.

A very promising model of treatment of bladder cancer using chitosan as a temporary destructor of the barrier function in the urothelium was developed by D.A. Zaharoff’s group combining immune stimulant interleukin 12 (IL-12) with chitosan (chitosan glutamate, 75–90% degree of deacetylation, 200–600 kDa) that allows the penetration of the drug into the urothelium. The four-times-repeated treatments with a combination of IL-12 and chitosan eliminated 90% of bladder tumours in experimental animals and provoked a memory response protecting the animals from bladder tumours for the rest of their life [44] (Figure 4). Further study of the same research group demonstrated the ability of chitosan-enhanced interleukin-12- chitosan (chitosan glutamate, 75–90% degree of deacetylation, 200–600 kDa) based therapies to engage adaptive immunity within the tumour itself as well as throughout the body, and strengthen the case for clinical translation of chitosan-interleukin 12 as an intravesical treatment for bladder cancer [45].

## 4. Chitosan in Treatment of Bacterial Infection of the Urinary Bladder

**Figure 5:**
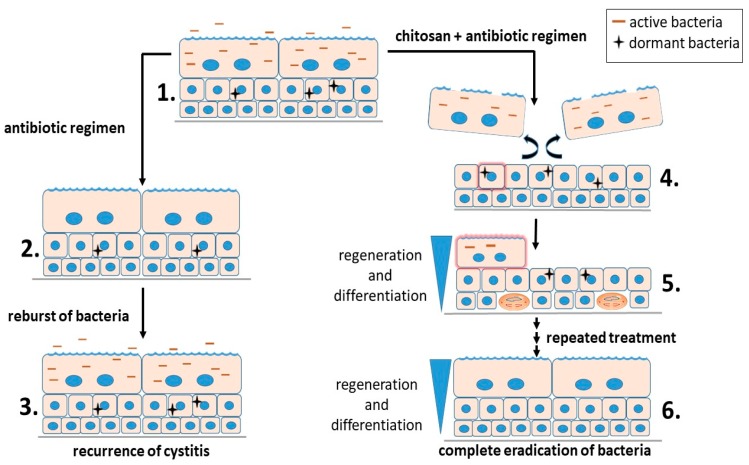
Two treatment regimens of infected urinary bladder epithelium. (**1**) Both active and dormant bacteria dwell in infected urothelial cells. (**2**) Antibiotic regimen efficiently clears active bacteria from both urine and host cells. However, immature urothelial cells retain dormant bacteria. (**3**) From the nests of dormant bacteria, new outbursts of bacteria appear periodically. (**4**) Treatment with a combination of chitosan and antibiotics induces a detachment of umbrella cells and activates differentiation of immature cells (red glowing cell). (**5**) Dormant bacteria reactivate in such differentiating cells (red glowing cell) and become again sensitive to antibiotics. Proliferating basal cells enable fast replacement of lost umbrella cells. (**6**) By repeated treatment with chitosan and antibiotics, bacteria become completely eliminated from urine and urothelial cells.

Urinary tract infections (UTI) include infections of the urethra (urethritis), bladder (cystitis), ureters (ureteritis), and kidney (pyelonephritis) [46]. Uropathogenic Escherichia coli (UPEC) is the primary agent of urinary tract infections. There is an estimated annual occurrence of over 8 million UTIs in the United States [47]. Nearly all patients with UTI are prescribed a regimen of antibiotics. The annual cost of UTI treatment in the United States is estimated at $2.14 billion [48]. Despite administration of antibiotics that clear the bacteria from urine, the probability that a patient will develop a second UTI within six months is 25%. In more than 50% of recurrent UTI episodes, the bacterial strains responsible for both the initial infection and the recurrence are genetically identical [49], which indicates the involvement of the intracellular bacterial reservoirs within the bladder epithelial cells from which these recurrences originate. UPEC can invade umbrella cells, as well as the underlying less differentiated urothelial cells. UPEC can rapidly multiply in the cytosol of umbrella cells, forming a biofilm-like assembly known as an intracellular bacterial community (IBC) [50]. The development of IBCs can enhance the ability of UPEC to prosper within the urinary tract, while being sequestered away from the immune system of the host [51]. The occasional exit of UPEC from umbrella cells enables the distribution of UPEC to the new uninfected urothelial cells of all differentiation stages. Within less differentiated urothelial cells, individual UPEC can enter a dormant state [50]. The quiescent nature and intracellular localization of these bacteria protects them from most antibiotic treatments. These quiescent intracellular UPEC reservoirs (QIRs) can persist for long periods of time in the absence of any clinical symptoms and with no signs of bacterial existence in urine [51]. Differentiation of immature urothelial cells hosting quiescent bacteria can trigger the resurgent growth of UPEC, causing the development and dispersal of IBCs and the reinitiation of clinical symptoms [46]. These issues urge the need for therapeutic strategies that effectively target both active and dormant stages of UTI. By inducing the exfoliation of the superficial layer of the urothelium, chitosan was shown to stimulate rapid regenerative processes and the reactivation and efflux of quiescent intracellular UPEC reservoirs. When combined with antibiotics, chitosan treatment (chitosan hydrochloride, 86% degree of deacetylation, 30–400 kDa) significantly reduced bacterial loads within the urine and also eradicated bacteria from the bladder wall and was proposed to be of therapeutic value to individuals with chronic, recurrent UTIs [52]. However, though a single treatment of chitosan followed by ciprofloxacin administration had a marked effect on reducing UPEC titters within the bladder, this treatment failed to prevent relapsing bacterial outbursts. Our recent study elucidates that after four repeated applications of chitosan chitosan (chitosan hydrochloride, 86% degree of deacetylation, 30–400 kDa) in combination with the antibiotic ciprofloxacin, a complete eradication of UPEC from the urinary tract was achieved with no relapsing bouts of bacteriuria and no lasting harm to the urothelium [53] (Figure 5).

## 5. Conclusions

Chitosan offers versatile biomedical applications in urinary bladder epithelial cells due to the capability of this polymer to transitorily abolish the barrier function of urothelium and consequently to enable better penetration of specific drugs to the deeper cell layers. Chitosan has a great potential for clinical application in treatment of bladder tumours as a supplementary system in combination with cytostatic and immunotherapy, and also as an auxiliary antimicrobial drug in treatment of uroinfections. Common in both treatments of bladder cancer and bacterial infection of the urinary bladder is the benefit of local topical application, where higher dosages can be applied and the systemic side effects of the drugs otherwise used by oral administration is omitted.
